# Surgical Stress Induces an Amplified Inflammatory Response in Patients with Type 2 Diabetes 

**DOI:** 10.1155/2013/910586

**Published:** 2013-01-21

**Authors:** Edward Lin, Nana Gletsu-Miller

**Affiliations:** ^1^Emory Endosurgery Unit Division of General and Gastrointestinal Surgery, Department of Surgery, Emory University School of Medicine, 1364 Clifton Road, Atlanta, GA 30322, USA; ^2^Department of Nutrition Science, College of Health and Human Sciences, Purdue University, West Lafayette, IN 47907-2059, USA

## Abstract

*Background*. Morbid obesity is believed to be an extreme of the metabolic spectrum. Moreover, diabetes is hypothesized to be associated with a chronic inflammatory state that is not observed in nondiabetic healthy individuals. We investigated the differences in expression of inflammatory cytokines induced by surgical stress between diabetic and nondiabetic individuals. *Method*. 39 morbidly obese patients undergoing laparoscopic Roux-en-Y gastric bypass (9 with type 2 diabetes mellitus) were compared with 8 nonobese euglycemic patients undergoing laparoscopic antireflux surgery. Cytokine levels for IL-6, IL-10, and IL-18 were measured 15 minutes before surgery and immediately after surgery. *Results*. IL-6 and IL-10 levels were elevated from baseline following surgery, but morbidly obese patients exhibited a much higher elevation than lean patients. Individuals with type 2 diabetes had the most pronounced IL-6 and IL-10 elevations. Baseline IL-18 levels were significantly higher in diabetic patients compared with nondiabetic or lean patients. However, IL-18 levels were not changed in response to surgery. *Conclusions*. Diabetes and morbid obesity are associated with augmented cytokine expression in response to surgical trauma that is several folds higher than in nonobese euglycemic patients. Diabetic patients exhibit a chronic elevation in IL-18 that is not changed by surgical stress.

## 1. Introduction 

Surgical stress induces an appropriate systemic inflammatory response that has been extensively characterized [1]. The initial inflammatory response is inherently designed to eradicate microorganisms, promote healing following injury, and restore homeostasis. However, perpetual or excessive inflammatory response may overwhelm the compensatory capacities of the host, eventuating in multiple-organ failure and patient demise. Cytokines, produced by immunoactive cells, are critical mediators of the inflammatory process and the response of tumor necrosis factor-*α*, interleukin- (IL-) 6, and IL-10 have all been described in the context of sepsis, surgical injury, and trauma [2].

 Patients with severe obesity also have significant comorbidities that include, but are not limited to, coronary artery disease, type 2 DM, and pulmonary diseases. These patients represent an extreme in metabolic and physiologic dysfunctions, which under conditions of stress may portend deleterious outcomes. Indeed, there is mounting evidence suggesting that the condition of severe obesity is a chronic systemic inflammatory state with cytokine mediators contributing to the pathophysiology of the associated comorbidities specifically pertaining to type 2 DM [3, 4]. However, the systemic inflammatory response to surgery for patients with underlying chronic inflammation such as in morbid obesity is not clear.

 Due to the known perioperative risks associated with weight-loss surgery [5], there is intense interest in being able to identify markers of injury as a potential tool to predict perioperative outcomes. This study characterizes the acute alterations in circulating IL-18 levels, a newer proinflammatory cytokine, in a set of consecutive patients undergoing laparoscopic roux-en-Y gastric bypass (RYGB) and in lean patients undergoing laparoscopic antireflux operations (LAR) to determine if this cytokine is a marker of systemic inflammatory response in obese patients. The study further attempts to determine if patients with type 2 DM exhibit different responses. The acute response of IL-18 to surgical stress is compared to the responses of two better-characterized cytokines, IL-6 and IL-10.

## 2. Methods

### 2.1. Patients

Using a protocol approved by the Institutional Review Board (IRB), 39 consecutive morbidly obese patients (body mass index, BMI > 40 kg/m^2^) undergoing laparoscopic RYGB were enrolled into the study. Thirty (30) of these patients did not have clinical diabetes as defined by no use of insulin or oral antihyperglycemic medications and normal fasting blood glucose levels. The other nine (9) patients had type 2 DM.

Eight (8) consecutive nonobese (BMI < 27 kg/m^2^), euglycemic patients undergoing laparoscopic antireflux surgery (LAR) served as lean controls. 

### 2.2. Surgical Procedures

In brief, the RYGB creates a longitudinal gastric pouch with a 15-to-30 cc volume, and a 150 cm roux-limb that is anastamosed to the gastric pouch. The LAR surgery involved complete circumferential mobilization of the esophagus into the mediastinum and the formation of a 360-degree (Nissen) fundoplication wrap.

### 2.3. Specimen Handling

 Blood was collected into EDTA-tubes 15 minutes preoperatively in fasting patients and immediately placed on ice. Blood was again collected immediately postoperatively after skin closure. Plasma was separated out in a cold centrifuge and immediately frozen at −80°C until analysis. IL-6, IL-10, and IL-18 levels were measured using high-sensitivity ELISA (R&D, Minneapolis, MN) and analyzed using a Bio-Rad 550 microplate reader. The limits of detection were 0.7 pg/mL, 3.9 pg/mL, and 12.5 pg/mL, respectively.

### 2.4. Statistics

Data were analyzed by ANOVA and Newman-Keuls test and reported as mean ± SEM.

## 3. Results

### 3.1. Patient Body Mass Indices

 Lean patient BMI was 26 ± 1.2 kg/m^2^. Nondiabetic obese patients and diabetic obese patients BMIs were 45 ± 1.3 and 46 ± 1.7 kg/m^2^, respectively (*P* < 0.001 compared with lean patients).

### 3.2. Patient Outcomes

There were no significant differences in outcomes between the two surgical groups. There were no deaths or major complications requiring reoperations or intensive care unit utilization. One urinary tract infection and one port-site infection were documented in the RYGB group. The length of hospital stay for the RYGB and LAR groups were 2.1 ± 0.2 and 1.25 ± 0.4, respectively.

### 3.3. IL-6 Response

**Figure 1:**
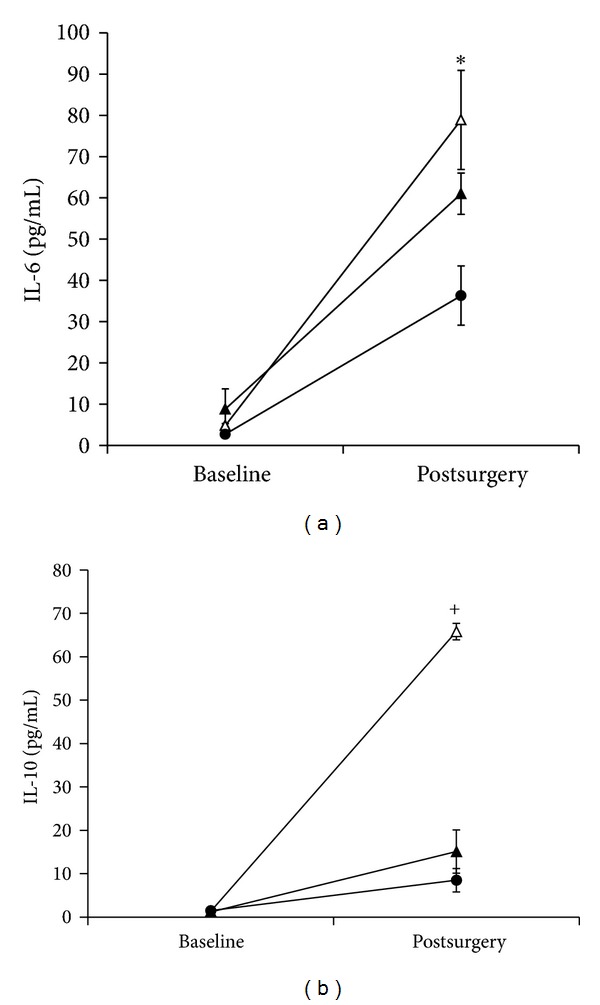
(a) Baseline and postsurgical IL-6 levels. (b) Baseline and postsurgical IL-10 levels. •: lean patients; ▲: nondiabetic obese; △: diabetic obese; *P < 0.01; ^+^ *P* < 0.001.

At baseline, all morbidly obese patients exhibited slightly higher IL-6 levels than lean patients (5.7 ± 1.3 and 2.7 ± 0.6 pg/mL, resp., *P* < 0.05). However, surgery induced the highest IL-6 response in diabetic obese patients (78.9 ± 12.3 pg/mL), a moderate response in non-diabetic obese patients (61.0 ± 5 pg/mL), and the lowest response in lean patients (36.3 ± 7.2 pg/mL) (*P* < 0.01) (Figure 1(a)).

### 3.4. IL-10 Response

 IL-10 levels were 1.5 ± 0.4, 1.2 ± 0.3, and 1.3 ± 0.3 pg/mL for non-diabetic obese, diabetic obese, and lean patients, respectively. Non-diabetic obese patients exhibited higher IL-10 postoperatively compared with lean patients (15.1 ± 5.0 and 8.5 ± 2.7 pg/mL, *P* = NS). However, like IL-6, surgery induced the highest IL-10 response in the diabetic obese patients (65.8 ± 1.9 pg/mL, *P* < 0.001) (Figure 1(b)).

### 3.5. IL-18 Response

**Figure 2:**
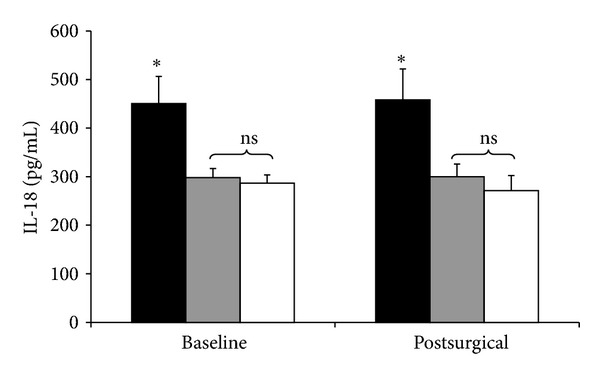
Baseline and postsurgical IL-18 levels. Black bars: diabetic obese patients. Grey bars: nondiabetic obese patients. White bars: lean patients. *P < 0.01.

Preoperative (baseline) IL-18 levels were significantly elevated in the diabetic obese patients (450.6 ± 56 pg/mL, *P* < 0.01), compared with non-diabetic obese patients (297.8 ± 19 pg/mL) and lean patients (286.6 ± 17 pg/mL). There was not a difference in baseline IL-18 between non-diabetic obese and lean patients. Postoperative IL-18 levels exhibited the same patterns, and remained virtually unchanged compared with baseline for all three groups (diabetic obese, 457.9 ± 64; non-diabetic obese, 300 ± 26; lean, 271.4 ± 31 pg/mL) (Figure 2).

## 4. Discussion

 This study describes novel observations regarding the differences in acute inflammatory responses of IL-6, IL-10, and IL-18 for severely obese patients with type 2 diabetes mellitus (DM) from the responses of lean patients.

 IL-18 is a proinflammatory cytokine that is released by various cell types but primarily by cells of the myelomonocytic lineage and is associated with human neutrophil, macrophage, and endothelial cell activation [6]. It shares biochemical features with IL-1*β* and acts in part by inducing interferon-gamma (IFN-*γ*) production by T cells. This cytokine augments vascular endothelial injury, specifically mediating atherogenic diseases [7], and potentiates immunocyte activation in scenarios such as acute lung injury [8]. Clinically, high IL-18 levels in the circulation are purported to be predictors of cardiovascular mortality, ischemic stroke outcome, and a marker for insulin resistance (type 2) diabetes mellitus (DM) [9–11]. While IL-18 is detectable in healthy human subjects, levels exhibited by critically ill patients with sepsis are nearly 10-fold higher [12].

 In all patient groups, IL-18 unexpectedly did not exhibit any changes from baseline in response to the stress of surgery. There is evidence that the acute expression of IL-18 is *selectively* induced, which is unlike the responses observed with other cytokine mediators such as IL-6 and IL-10 [13]. Critically ill patients with sepsis exhibited significantly higher levels of IL-18 compared with healthy subjects administered small doses of endotoxin [12]. In other studies, septic patients who died had higher IL-18 levels than survivors [14, 15]. It has been further demonstrated that IL-18 levels are significantly higher in patients with Gram-positive sepsis than in Gram-negative sepsis [16]. One can speculate that the higher IL-18 levels in diabetic obese patients at baseline may portend poorer outcomes should a surgical complication occur, but this is unproven.

 Outside of acute illness, higher IL-18 levels have been associated with chronic conditions such as heart failure, diabetic nephropathy, and inflammatory bowel disease [9, 11, 17]. However, in the context of severe obesity, whether IL-18 is a biomarker of comorbid conditions or a mediator of disease is unclear. Some have suggested that IL-18 levels correlate with visceral fat volume in women, and that a 10% weight loss significantly reduces IL-18 levels and improves cardiac contractility [18]. We have also observed that previously diabetic obese patients who present for various abdominal surgeries 12 months to 10 years after RYGB exhibit IL-18 levels that match those of nondiabetic patients. The present study did not show a baseline difference between lean patients and nondiabetic obese patients, but there was a significantly higher IL-18 level in severely obese patients with type 2 DM. These observations may suggest that it is IL-18, rather than obesity itself, that is associated with dysregulated insulin-glucose metabolism in patients with type 2 DM.

As expected for both IL-6 and IL-10, surgery induced a significant elevation in levels from baseline [19]. However, the stress of surgery induced a far greater elevation for diabetic obese patients than for nondiabetic patients. This is among the first such observation that captures the amplified inflammatory response to injury in patients with type 2 DM. At present, the clinical relevance of such an exaggerated response can only be inferred because of the modest number of patients and overall uniform outcomes in this study.

 There is an extensive pool of evidence suggesting that the chronic inflammatory state induced by severe obesity is responsible for the observed insulin resistance [20]. Indeed, proinflammatory cytokines such as IL-6 have been demonstrated to inhibit glucose transport in adipocytes [21, 22]. The inhibition of insulin metabolism is likely due to competing mechanisms in the postreceptor signaling pathways of the insulin receptor [23, 24].

 The role of IL-10 in type 2 DM is less clear. However, being an anti-inflammatory cytokine, it conceivably counteracts the insulin-resistance effects of proinflammatory mediators. In a study that sampled the blood of over 700 elderly inhabitants in the city of Leiden, subjects with the lowest IL-10 production from whole-blood assays were more likely to have type 2 DM than those with the highest IL-10 levels [25]. Perhaps, the odds of developing insulin resistant diabetes may very well be affected by the ratio of proinflammatory to anti-inflammatory mediators. Conversely, such a ratio may have potential utility for predicting the likelihood that type 2 DM will resolve after weight-loss surgery.

 In this study, the IL-10 response in diabetic obese patients was 50-fold from baseline after surgery, but only 11-fold higher in the non-diabetic obese patients. This may be an adaptive mechanism in severely obese patients with diabetes to compensate for the augmented proinflammatory response following surgical stress and avert any overwhelming systemic inflammation [26].

 Where is the origin of these inflammatory cytokine mediators? Clearly, the immunoactive cells such as monocytes, neutrophils, and lymphocytes all participate to orchestrate the inflammatory response. However, it is important to appreciate the adipose tissue as a potential source of inflammatory mediators, particularly when cytokine levels are reduced following weight loss—surgically induced or otherwise [4, 18, 27]. Stress hormones can induce IL-6 production from adipocytes [28]. In addition, migrating macrophages and reticuloendothelial cells as well as adipocyte precursors residing within adipose tissues are very potent sources of inflammatory mediator release and contribute to the development of obesity-related insulin resistance [29]. There is also evidence that the concentration of macrophage infiltration into adipose tissue is associated with BMI and adipocyte size [30]. These studies, taken together, imply that fat volume distributed in the subcutaneous or visceral compartments are immunologically active and indeed contribute to the inflammatory response of abdominal surgery proportional to its inflammatory cell concentration. In our preliminary experience (unpublished data), RYGP patients returning within 16 months for subsequent surgical procedures after significant weight loss (e.g., cholecystectomy, major cosmetic surgery) exhibit a tempered rise in inflammatory cytokines compared with the response from the original weight-loss surgery.

 This study provides global demonstration that adiposity can contribute to the inflammatory response induced by surgery. Moreover, patients with type 2 DM have amplified proinflammatory responses compared to their non-diabetic obese counterparts. Issues that remain unresolved are (1) whether the exaggerated inflammatory mediator release in severely obese patients are potential predictors of poorer surgical outcomes and (2) the relative contributions of different body fat compartments to the systemic inflammatory response and possibly to insulin resistance.
